# Clinical Studies Using Intranasal Therapies for Parkinson’s Disease: A Review

**DOI:** 10.34172/apb.025.46111

**Published:** 2025-12-27

**Authors:** Ramin Vahidi, Mahsa Bukanian, Maryam Kachooeian

**Affiliations:** ^1^Student Research Committee, Alborz University of Medical Sciences, Karaj, Iran; ^2^Department of Pharmaceutics, Faculty of Pharmacy, Alborz University of Medical Sciences, Karaj, Iran

**Keywords:** Intranasal drug delivery, Parkinson disease, Off periods, Spray

## Abstract

Intranasal delivery is a method of administering medications through the nasal cavity. It offers several advantages, such as rapid absorption, bypassing first-pass metabolism, direct nose-to-brain transport and localized effects. These benefits make it a promising approach for drug delivery in Parkinson’s disease, a progressive neurological disorder characterized by the degeneration of nerve cells in the brain. This review evaluates the efficacy and safety of intranasal delivery for Parkinson’s disease treatment. Several studies on intranasal apomorphine reported rapid clinical response, improved UPDRS motor scores, tapping scores, and median Webster’s scores, suggesting its effectiveness as a rescue therapy during "off" states. Intranasal recombinant erythropoietin was well tolerated and showed cognitive benefits. intranasal glutathione was safe and showed better bioavailability. Intranasal insulin improved cognitive performance without hypoglycemia, indicating a localized effect. Intranasal cholecystokinin and ipratropium bromide did not show significant benefits. Intranasal desmopressin is a safe and effective medication for nocturnal polyuria in Parkinson disease. Intranasal transplantation of neural stem cells is safe and is associated with functional improvement. Finally, Rivastigmine nasal spray offered better bioavailability and fewer side effects compared with conventional forms. The most common adverse effect was mild transient nasal or throat irritation. This review highlights the potential applications, efficacy, and side effects of various intranasal medications for Parkinson’s disease and proposes using new interventions for future studies. The general benefits of nasal administration for Parkinson’s disease treatment include localized effects, fewer side effects, faster onset of action, improved bioavailability, and enhanced therapeutic effectiveness.

## Introduction

 Parkinson’s disease (PD) is a progressive neurodegenerative disorder characterized primarily by motor symptoms including bradykinesia, rigidity, tremor, and postural instability and a group of non-motor symptoms such as Autonomic dysfunction and Behavioral changes also play a critical role in disease burden.^1^ progressive loss of dopaminergic neurons in the substantia nigra, leads to striatum dopamine depletion which is widely recognized as the primary pathological sign of PD.^2^ Although current treatments such as oral levodopa and dopamine agonists have transformed clinical management, limitations in their pharmacokinetic profiles, including variable absorption, delayed onset are significant challenges^3^ which can be improved by new formulations.

 Based on the challenges mentioned, alternative drug delivery methods have attracted considerable interest. Although the surface area of the olfactory epithelium and administrated volume are low^4^ Intranasal administration is a promising route for PD therapies due to its potential to bypass blood–brain barrier by olfactory and trigeminal nerve pathways.^5-8^ This route can provide rapid drug absorption, faster onset of action, and lower systemic exposure compared to traditional oral or injectable dosage forms^9,10^ and its appropriate for daily usages.^7^ Beside well known intranasal solutions there are various formulations such as Mucoadhesive agents^11^, Nanoparticles^12^, gels^13^ and lipid based systems.^14^ These features are favorable in the context of PD where faster onset of action in “off” episodes and reduction of systemic effect can improve patient outcomes.

 This review is conducted to evaluate current clinical evidence on intranasal drug delivery for Parkinson’s disease treatment, asking can intranasal delivery be a better way of administration than conventional therapies? This review addresses key findings regarding the clinical efficacy, safety, and tolerability of intranasally administered agents, ranging from dopaminergic drugs such as apomorphine to emerging agents including insulin, glutathione, erythropoietin, and cell-based therapies. By evaluating numerous studies, we seek to identify both the benefits and limitations of this approach. For this review, we searched PubMed, Web of Science, Scopus, and Google Scholar databases using relevant keywords. The search yielded 2,103 records, of which twenty-three were related to our topic.

 In summary, this review provides an assessment of available clinical data findings on intranasal therapy in PD, offering insight into its potential to overcome the shortcomings of conventional drug delivery systems. In the following section, we will delve into each study using intranasal formulations for Parkinson’s disease treatment, categorized into Motor Symptom Management, Cognitive and Other Non-Motor Symptom Management, Neuroprotection and Oxidative Stress Reduction, and Specific Non-Motor Symptom Management. Finally in the conclusion section, we will sum up the efficacy of each treatment and give suggestions for future studies.

## Motor Symptom Management

 Motor symptoms of Parkinson disease include rest tremors, rigidity, brady dyskinesia and loss of postural reflexes.^15^ dissabling Parkinson patient from daily activity. Using a fast acting or long-acting agent can improve Parkinson disease patients’ quality of life.

###  Intranasal Apomorphine

 Apomorphine is a short-acting D_1_- and D_2_-like receptor agonist. apomorphine has dopaminergic side effects such as nausea, hypotonia, as well as administration site reactions which can cause discontinuation of treatment.^16^ here are some clinical studies looking for efficacy of intranasal apomorphine:

 In one study comparing intranasal and subcutaneous apomorphine,Intranasal apomorphine showed bioavailability of 45% relative to subcutaneous administration. The time to ‘on’ and the duration of ‘on’ were comparable between intranasal and subcutaneous routes, and no statistically significant differences were observed in pharmacokinetic parameters or clinical outcomes between the two administration methods.^9^ In Another studyIntranasal apomorphine improved Unified Parkinson’s Disease Rating Scale(UPDRS) motor scores compared with baseline (*P* < 0.04). The mean latency to response was 11 minutes. duration of effect was 50 minutes, and 72–81% of doses were rated effective versus 3–22% for placebo. Off-hours per day were reduced with apomorphine and trimethobenzamide (*P* = 0.02). Tapping and Webster’s scores improved in one treatment arm (*P* < 0.04), and diary-based measures of response magnitude and percentage of effective doses were significantly better than placebo (*P* = 0.01–0.03).^17^ in other study usingInhaled apomorphine, in-clinic UPDRS 3 scores compared with placebo group (19.5 vs 9.9; least squares mean difference 8.4, *P* = 0.023) was significantly improved. Latency to ‘on’ was significantly shorter with apomorphine (8.1 minutes vs 13.1 minutes; *P* < 0.0001), and 64.6% of ‘off’ episodes were aborted versus 11.1% with placebo (*P* < 0.0001). The odds of achieving ‘on’ or ‘partial on’ within 40 minutes were higher with apomorphine (odds ratio 4.4; *P* = 0.045). Reduction in at-home daily ‘off’ time (139.8 vs 68.0 minutes) was not statistically significant (*P* = 0.078). Other measures, including daily ‘on’ time and number of ‘off’ episodes, showed no significant differences.^18^ A phase two clinical study of Inhaled apomorphine showed significant improvement of UPDRS 3 scores compared with placebo (26.8 vs 14.9; least squares mean difference 11.6, *P* = 0.016). A higher proportion of apomorphine-treated patients converted from ‘off’ to ‘on’ compared with placebo (81.3% versus 46.7%, *P* = 0.025). Time to ‘on’ and duration of ‘on’ were numerically shorter and longer with apomorphine, but these differences were not statistically significant (*P* = 0.461). No dose-related effects were observed. Inhaled apomorphine can be a good candidate for replacement with intermittent subcutaneous injections.^19^ Another study usingInhaled apomorphine showed dose-dependent efficacy in the proportion of patients switching from ‘off’ to ‘on’ (0% at 0.2 mg, 50% at 0.5 mg, 33.3% at 0.8 mg) versus 16.7% for placebo. Duration of ‘on’ ranged from 0–40 minutes for apomorphine versus 20 minutes for placebo. UPDRS 3 upper limb scores suggested improvement at 0.8 mg. However, none of the outcomes, including proportion switching to ‘on’, time to ‘on’, duration of ‘on’, or proportion achieving ‘on’ or ‘partial on’, reached statistical significance. Inhaled apomorphine is safe and well tolerated, but efficacy at these doses was limited. ^20^ In a three-part phase 1 study on PD patients, apomorphine inhalation reduced MDS-UPDRS III scores at 10 minutes compared with placebo: 10.7 (2 mg), 12.8 (3 mg), and 10.3 (4 mg) versus 4.8 for placebo. The proportion of patients achieving full ‘on’ within 45 minutes was 17% (2 mg), 50% (3 mg), and 83% (4 mg) versus 0% for placebo. This intervention seems to be fast and well tolerated^21^. In this study apomorphine was rapidly absorbed, with median Tmax of 1–2 minutes. At 4 mg, MDS-UPDRS III scores showed mean reductions of 6.8 points at 10 minutes and 6.1 points at 30 minutes post-dose. In the crossover study, 50% of patients achieved ‘on’ at 10 minutes after 4 mg apomorphine, compared with 0% for placebo.^22^ At last study Intranasal apomorphine significantly reduced mean daily off-hours from 5.37 at baseline to 0.56, compared with 0.72 for subcutaneous administration. Mean onset was 8.25 minutes, and mean duration of response was 57.5 minutes. UPDRS motor scores improved, especially for bradykinesia and rigidity without increasing dyskinesia. Off-hours reductions were near-significant versus baseline (*P* < 0.0679) and favored intranasal versus subcutaneous (*P* < 0.069).^23^

###  Human Neural Stem Cells

 In this study Intranasal human neural stem cellANGE-S003 was well endured with no serious adverse events or MRI abnormalities. Functional improvements appeared by month 3, peaked at month 6, and persisted through month 12. The mean MDS-UPDRS total score reduction was 19.9 points at month 6 (*P* < 0.001), independent of dose, indicating sustained symptomatic improvement in advanced PD.^24^

 a promising preclinical study investigated magnetically targeted intranasal administration (MTCD) of alginate-coated, nanoparticle-labeled human olfactory ectomesenchymal stem cells (OE-MSCs) in a 6-hydroxydopamine-induced Parkinson’s disease rat model. MTCD enhanced stem cell delivery, improved motor performance, increased dopaminergic neuron survival, and upregulated Dopamine transporter (DAT), Nurr1, and paired-like homeodomain transcription factor 3 (TH) expression. MRI tracking confirmed robust targeting (*P* < 0.0001), with preserved cell viability. These findings highlight MTCD as a promising, non-invasive therapeutic strategy in future studies for Parkinson’s disease.^25^

## Cognitive and Non-Motor Symptom Management

 depression, apathy, anxiety, sleep disorders and sensory abnormalities are among the most frequent cognitive and non-motor symptoms.^15^ in this section we evaluate insulin,NeuroEPO, Cholecystokinin and Rivastigmine on these symptoms.

###  Intranasal Insulin

 Insulin resistance in brain may play a role in the pathophysiology of PD. Using antidiabetic agents has gained interest in being evaluated as a PD treatment.^26^

 In a study using Intranasal insulin. verbal fluency improved by 5.6% compared with a 6.4% decrease in placebo (*P* = 0.02). Hoehn and Yahr scores improved significantly in the insulin group versus placebo. MDS-UPDRS Part III (motor) scores improved in the insulin group (*P* = 0.02 vs. baseline) but not in placebo. No significant changes were observed in Montreal Cognitive Assessment (MoCA), Beck Depression Inventory (BDI), or gait measures. Treatment showed an acceptable safety profile.^10^

 Also, two Phase 2 trials are investigating intranasal insulin in PD patients (NCT04687878; NCT04251585) searching for its efficacy on Motor and Non-motor Symptoms in Parkinson’s Disease Patients.

###  NeuroEPO

 Recombinant erythropoietin (EPO) is an erythropoiesis-stimulating agent increased in anemia, it is produced and manufactured in Cuba (iorEPOCIM, CIMAB S.A, Havana, Cuba) has neuroprotective properties. NeuroEPO is a nasal formulation of recombinant EPO containing low quantity of sialic a without hematopoietic effects. It has shown neuroprotective effects in animals.^27^

 In a study Both recombinant human erythropoietin (rhEPO) and intranasal neuroEPO demonstrated cognitive improvements in Parkinson’s disease patients. rhEPO improved Dementia Rating Scale (DRS) scores in all patients after treatment (z = 2.84, *P* = 0.004). Intranasal neuroEPO enhanced phonological verbal fluency at week 1 (z = 2.2, *P* = 0.02) and semantic fluency at six months (z = 2.13, *P* = 0.03). DRS scores improved in week 1 (*P* = 0.01, z = 2.5) and six months (*P* = 0.005, z = 2.8), while Frontal Assessment Battery (FAB) scores and Rey Complex Figure memory and copy subtests also showed significant improvements in one week and six months’ post-treatment. No major adverse events were reported, though between-group differences for neuroEPO versus placebo were not significant, indicating possible placebo effects.^28^ In this study intervention consisted of weekly intranasal administrations of NeuroEPO (1 mL at 1 mg/mL) for 5 weeks in Parkinson’s disease patients. NeuroEPO was acceptable, with mild and transient adverse effects reported in 20% of patients treated versus 9.1% in placebo. There were no significant changes in blood pressure and biochemical parameters. Statistical analyses confirmed no differences in adverse event frequency or laboratory measures (*P* > 0.05). The study demonstrated short-term safety of NeuroEPO over five weeks.^27^ In another study intranasal NeuroEPO was administered at 1 mg weekly for 5 weeks in Parkinson’s disease patients without cognitive impairment. NeuroEPO significantly improved cognition (*P* = 0.006) with 66% of the improvement mediated by quantitative electroencephalogram (qEEG) changes (*P* < 0.0001). Mediation analysis confirmed both strong indirect and direct effects (*P* = 0.002). The treatment demonstrated good tolerability, with no major safety concerns emphasized. These findings indicate that intranasal NeuroEPO enhances cognition in PD, primarily through EEG-mediated mechanisms.^29^

###  Rivastigmine

 Rivastigmine is a slow-reversible, noncompetitive carbamate cholinesterase inhibitor which is approved for mild to moderate Alzheimer’s disease treatment.^30^

 This study evaluated rivastigmine nasal spray’s bioavailability and safety. Participants received rivastigmine IV (1 mg) and nasal spray (3.126 mg). nasal spray demonstrated significant absolute bioavailability (F = 0.62, SD 0.15, *P* < 0.001) with an absorbed dose of 2.0 (0.6) mg, Tmax 1.1 (0.5) h, Cmax 6.9 (2.0) ng/mL, and metabolite ratio 0.78 (0.19). Two mild, transient adverse events (nasal/throat irritation) were observed resolving within 20 minutes. no serious events reported. Bioavailability exceeded historical oral (0.36) and transdermal (0.30–0.56) values.^31^

## Neuroprotection and Oxidative Stress Reduction

###  Cholecystokinin

 Cholecystokinin (CCK) is a neuropeptide which supports memory, modulates dopamine signaling, and provides neuroprotection in Alzheimer’s and Parkinson’s diseases via mitochondrial, autophagic, and anti-inflammatory pathways.^32^

 In this study intervention was a single intranasal administration of cholecystokinin-8 (CCK-8, 25 µg) in a double-blind, placebo-controlled, crossover design. In Parkinson’s disease patients, CCK-8 significantly delayed N2/P3 latencies during cognitive testing, indicating impaired attentive processing, while in healthy controls, latencies shortened and P3 amplitude increased, reflecting facilitation. No motor improvements were observed. These results suggest CCK-8 has cognitive, non-dopaminergic effects, potentially deleterious in PD, with no demonstrated motor efficacy.^33^

###  Intranasal Glutathione

 In Parkinson’s disease Redox dysfunction and neuro-oxidative stress. lowered glutathione (GSH) levels and GSH/GSSG imbalance are linked to mitochondrial dysfunction, neuroinflammation, and neurodegeneration. but it remains unclear whether GSH dysregulation is caused by or is consequence of PD.^34^ studies on intranasal glutathione are as follows:

 In this study, intranasal glutathione was administrated 3 times per day for one month in 3 groups(intranasal saline as Placebo, intranasal glutathione 300 mg/day as Low-dose group and intranasal glutathione 600 mg/day as High-dose group).Treatment was safe and acceptable to patients, with high adherence ( > 80%).UPDRS scores showed non-significant trends toward improvement in both glutathione arms compared with placebo.^35^ In an retrospective survey of intranasal reduced glutathione (inGSH) across conditions including Parkinson’s disease, multiple chemical sensitivity, and allergies/sinusitis found that among 66 users (mean age 56.8 years), 78.8% reported a positive experience and 62.1% perceived health benefits, such as symptom improvement, increased well-being, and higher energy. Mild adverse effects occurred in 12.1% (nasal irritation, headaches, epistaxis). No adverse events were reported in PD participants and 42.9% of them noted benefits including energy and symptom relief. Results suggests that inGSH is generally safe, well-tolerated, easy to administer, and potentially beneficial, supporting further controlled studies.^36^ In another study a single 200 mg dose of intranasal reduced glutathione (inGSH) significantly increased brain GSH levels in 15 mid-stage Parkinson’s disease patients, as measured by Meshcher-Garwood point resolved spectroscopy (MEGA-PRESS) and proton magnetic resonance spectroscopy (H-MRS). Elevations were not observed at 8 minutes but were sustained from 16 to 60 minutes post-dose, peaking around 45–60 minutes. Mild nasal irritation occurred in two participants. One-way repeated measures ANOVA confirmed significant time-dependent increases (F (6,84) = 12.34, *P* < 0.001). These findings indicate that intranasal GSH can rapidly and safely augment CNS GSH.^37^ In a Phase IIb study 45 mid-stage Parkinson’s disease patients received intranasal reduced glutathione ((in)GSH) at 300 or 600 mg per day doses or placebo for 3 months. followed by a 1-month washout. 600 mg/day intranasally administered glutathione in Parkinson’s patients showed within-group improvements in total UPDRS (–4.6, *P* = 0.0025) and motor scores (–2.2, *P* = 0.0485) as well as significant reductions in nonmotor symptoms (NMSS –10.17, *P* = 0.0217). No alterations were observed in blood or CNS oxidative biomarkers. One case of cardiomyopathy occurred in the high-dose group and was possibly treatment-related. high-dose GSH demonstrated modest symptomatic benefits without clear biomarker changes and superiority over placebo was not established.^38^

## Specific Non-Motor Symptom Management

 Sialorrhea and nocturia are being investigated in this section. majority of PD patients suffer from non-motor salivary symptoms such as sialorrhea and xerostomia impairing patient oral health and overall quality of life.^39^ other common complication is nocturia. Nocturia in these patient can be as result of reduced bladder capacity or nocturnal polyuria.^40^

###  Ipratropium Bromide

 Sialorrhea is a non-motor symptom in advanced Parkinson’s disease (PD). This study hypothesized Ipratropium Bromide, an anticholinergic agent that can reduce drooling in PD patients. Conventional option is systemic anticholinergics which frequently cause side effects. ipratropium bromide spray used sublingually did not significantly reduce saliva weight compared with placebo (*P* > 0.05). Secondary measures showed mild subjective improvements, but no significant differences were observed between treatment arms. The spray was well tolerated.^41^

###  Desmopressin

 Desmopressin (dDAVP) is a synthetic analog of arginine vasopressin which enhances antidiuretic potency, minimal pressor activity, and a prolonged effect. Its primary use is as the treatment of choice for central diabetes insipidus. It can be administered intranasally or parenterally.^42^

 In this study using intranasal desmopressin in Parkinson disease patients significantly reduction in nocturnal voids at the higher dose was observed. mean nocturnal voids decreased from 2.67 ± 0.14 at baseline to 1.51 ± 0.14 at the 20 μg dose (*P* = 0.0431; *P* = 0.011). Safety was generally acceptable Patients reported symptomatic benefits, and four continued long-term therapy, although one case of hyponatremia occurred, which resolved upon discontinuation.^43^

###  Overall Adverse Events

 Across the studies, the most reported adverse event was mild and transient nasal or throat irritation, which was generally well tolerated and did not necessitate treatment discontinuation, making intranasal formulation more acceptable for patients and better compliance (Table 1).

**Table 1:** summary of intranasal interventions used for Parkinson disease treatment.

**Category**	**treatment**	**Key Findings**	**Adverse Events**	**Study Limitations**
Motor Symptom Management	Apomorphine	Rapid onset (1–18.1 min); UPDRS motor score reductions (up to 26.8 points, *P* = 0.016–0.023); reduced daily ‘off’ time (up to 139.8 min, *P* = 0.078); 64.6% ‘off’ episodes aborted (*P* < 0.0001); 81.3% converted to ‘on’ (*P* = 0.025).^9,17-20,22,23^	Mild to moderate nasal/throat irritation, nausea, somnolence, vestibulitis; disabling irritation in 3 patients; 1 unrelated serious AE.^17-20,22,23^	Small sample sizes (n = 4–55); variable dosing (0.2–5.75 mg); lack of significant differences in some outcomes (*P* > 0.05); no dose-related effects in some studies.^9,19,20,23^
	Intranasal Transplantation of Human Neural Stem Cells (ANGE-S003)	Improvement was seen in MDS-UPDRS total score for 16 patients at all time points (*P* < 0.001), starting month 3, sustained to month 12. max reduction 19.9 points at month 6 (95% CI 9.6-30.3, *P* < 0.001). no association with dose levels (1.5M, 5M, 15M cells, four administrations). no mass formation on MRI. feasible and well-tolerated.^24^	14 adverse events in 7/18 patients over 12 months. no serious or related to treatment.^24^	small sample size (n = 18) and dose-escalation without placebo control^24^
Cognitive and Non-Motor Symptom Management	insulin	Improved verbal fluency (5.6%, *P* = 0.02); UPDRS motor score improvement (*P* = 0.02); Hoehn and Yahr Scale (HY) score improvement vs. placebo.^10^	1 unrelated AE leading to withdrawal; no hypoglycemia or serious AEs.^10^	Small sample size (n = 14); pilot study; short duration (4 weeks); 1 patient with possible multiple system atrophy (MSA) included.^10^
	NeuroEPO	Enhanced cognition (*P* = 0.006, 66% EEG-mediated); improved phonological fluency (*P* = 0.017); trends in DRS, FAB, Rey figure. ^27-29^	20% had mild, transient nausea/vomiting; 9.1% placebo had polyuria, nasal irritation; no significant lab changes.^27^	Small sample sizes (n = 25–26); short duration (5 weeks); placebo influence in some outcomes.^27-29^
	Cholecystokinin (CCK-8)	Delayed N2/P3 latencies in PD (*P* < 0.05); no motor improvements (*P* > 0.1); facilitated cognition in healthy controls.^33^	No major AEs reported; possible placebo influence.^33^	Small sample size (n = 13); single-dose design; limited generalizability.^33^
	Rivastigmine	High bioavailability (F = 0.62, *P* < 0.001); absorbed dose 2.0 mg; potential for dementia treatment.^31^	Two mild, transient nasal/throat irritation events; no serious AEs.^31^	Small sample size (n = 8); healthy subjects only; no PD-specific data.^31^
Neuroprotection and Oxidative Stress Reduction	Glutathione	Increased CNS GSH levels (*P* < 0.001); UPDRS trends (–5.3 high-dose, –4.3 low-dose); NMSS improvement (*P* = 0.0217); 57.1% PD patients reported benefits.^35-37,44^	Mild nasal irritation (2 patients); 1 pruritus exacerbation; 1 possible cardiomyopathy; 12.1% had irritation, headaches, epistaxis.^35-37,44^	Small sample sizes (n = 15–45); robust placebo response; no between-group differences; low survey response rate (23.3%).^35-37,44^
	Cholecystokinin (CCK-8)	Delayed N2/P3 latencies in PD (*P* < 0.05); no motor improvements (*P* > 0.1); facilitated cognition in healthy controls.^33^	Not specified in detail; no serious AEs implied.^33^	Small sample size (n = 13); single-dose design; limited generalizability.^33^
Specific Non-Motor Symptom Management	Ipratropium Bromide	No significant saliva weight reduction (*P* > 0.05); mild subjective improvements.^41^	1 possible nosebleed; no serious AEs.^41^	Small sample size (n = 15); no objective efficacy; short duration (2 weeks).^41^
	Desmopressin	Reduced nocturnal voids (1.51 at 20 μg, *P* = 0.011); patient-reported benefits.^43^	1 case of hyponatremia (resolved); no BP changes.^43^	Small sample size (n = 5 completers); open-label; short duration (2 weeks).^43^

## Discussion

 In this review we assessed intranasal interventions used in Parkinson disease patients (Table 2). Intranasal apomorphine, achieved faster onset of action (Tmax 1–2 minutes vs. 30 minutes for subcutaneous) and significant efficacy, with UPDRS motor score improvements (*P* = 0.016–0.023) and decreased ‘off’ episode rates (64.6% vs. 11.1%, *P* < 0.0001) compared to placebo.^9,18,19,22^. Intranasal insulin improved verbal fluency and motor scores (*P* = 0.02), suggesting potential cognitive and motor benefits.^10^ Glutathione increased CNS levels (*P* < 0.001) with trends toward UPDRS improvement, though placebo responses limited significance ^35,37,44^ These outcomes possibly support the role of olfactory and trigeminal pathways in bypassing the blood–brain barrier^5,6^. NeuroEPO enhances cognition via EEG-mediated mechanisms (*P* = 0.006). Human neural stem cells achieved sustained MDS-UPDRS reductions (*P* < 0.001).^24,29^ Desmopressin reduced nocturnal voids (*P* = 0.011). Ipratropium bromide showed no significant saliva reduction. Cholecystokinin impaired cognitive processing in PD patients (*P* < 0.05).^33,41,43^ Rivastigmine nasal spray demonstrated high bioavailability (F = 0.62, *P* < 0.001), suggesting its efficient CNS delivery.^31^

**Table 2:** study characteristics

**Study Reference**	**Intervention**	**Study Design**	**Population**	**Measurements **	**Key Results**	**Safety/Tolerability**
^9^	Intranasal and subcutaneous apomorphine	Comparative pharmacokinetic study^9^	Seven patients with Parkinsonism and 'on-off' problems	Pharmacokinetics (absorption kinetics, bioavailability, Tmax, lag time, elimination half-life) and time to 'on', duration of 'on'	Intranasal bioavailability 45% compared to subcutaneous; Tmax 23 min intranasal vs. 18 min subcutaneous; elimination half-life 31 min intranasal vs. 27 min subcutaneous; rapid absorption for both	No serious adverse effect mentioned
^17^	Intranasal apomorphine spray with or without trimethobenzamide antiemetic, up to three doses/day for 2 weeks/period	Double-blind, placebo-controlled crossover trial (2x2 factorial design)	Nine patients (7 completed) with advanced levodopa-responsive PD and motor fluctuations	Change in UPDRS motor score; secondary: tapping/Webster scores, diary measures (% effective doses, off-hours)	Significant reduction in UPDRS motor score with active apomorphine; latency to onset 11 min, duration 50 min; nausea in one patient; nasal irritation disabling in three, mild in two	Nausea from apomorphine in one patient; nasal irritation limiting in 3 patients; well tolerated with trimethobenzamide
^18^	Inhaled apomorphine (escalating doses:1.5, 2.5, 3.5, 4.5 mg with mean dose 2.3 mg)	Randomized, double-blind, placebo-controlled, parallel-group study (clinic and home-based)	Fifty-five patients with PD and 'on-off' fluctuations; mean age 65.6 years (47-79); mean disease duration 12 years (5-22)	Improvement in UPDRS Part 3 at highest dose; daily off time reduction; time to on; proportion turning on	UPDRS 3 improvement 19.5 points with apomorphine vs. 9.9 placebo (significant); off time reduced by 139.8 min vs. 68.0 min (not significant); onset 8.1 min vs. 13.1 min (significant); 64.6% episodes reversed vs. 11.1%	Adverse events in 36% apomorphine vs. 20% placebo; no serious AEs related to drug
^19^	Inhaled dry powder apomorphine (VR040) at 1.5, 2.3, 3.0, 4.0 mg doses	Double-blind, controlled with placebo, dose-ranging study	Forty-seven PD patients. mean age 60.6 years	UPDRS Part 3 response at highest dose	UPDRS 3 improvement 26.8 points with VR040 versus 14.9 with placebo, rapid onset at 10 min, rapid absorption (2-7 min)	No serious AEs related to drug; one unrelated Serious Adverse Event** (**SAE) was constipation and it is well tolerated
^20^	Inhaled apomorphine (0.2, 0.5, 0.8 mg doses)	Phase IIa randomized, double-blind, placebo-controlled study	Twenty-four patients with established PD and 'off' periods	Proportion switching from off to on and time from off to on	No significant increase in proportion turning on overall (5/12 at higher doses versus 1/6 with placebo); time to on 10-40 min versus 20 min placebo; rapid absorption (peak 1-3 min)	Safe and well tolerated at tested doses; no serious AEs
^21^	Inhaled apomorphine up to 4 mg in PD patients (2, 3, 4 mg)	Randomized trial (Part A: healthy volunteer (HV) crossover; Parts B/C: ascending dose in HVs and PD patients)	Part A: 8 HVs; Part B: 16 HVs; Part C: 25 PD patients with morning off	Safety, PK, efficacy (MDS-UPDRS III reduction; full on within 45 min)	Rapid absorption (Tmax 2 min); MDS-UPDRS III reduction 10.7-12.8 points at 10 min versus 4.8 for placebo); full on: 17% (2 mg), 50% (3 mg), 83% (4 mg) vs. 0% placebo	Well tolerated up to 3 mg in HVs, 4 mg in PD; mild transient throat irritation; AEs limited dose in HVs
^22^	Inhaled apomorphine at 2, 3, 4 mg doses up to three times daily	Part A: Multiple ascending dose in PD patients; Part B: Double-blind crossover in PD patients	Part A: 26 (24 completed) PD patients; Part B: 9 (8 completed) PD patients with off periods	Safety, PK (multiple dosing); efficacy (MDS-UPDRS III, on/off state)	Rapid absorption (Tmax 1-2 min); MDS-UPDRS III reduction 6.8-6.1 points at 10-30 min (placebo-corrected); 50% turned on at 10 min with 4 mg versus 0% with placebo	Relatively well tolerated; mild transient throat irritation and cough most common
^23^	Intranasal apomorphine (1 mg per puff, metered-dose nebulizer	Open-label study	4 patients with idiopathic PD and disabling on-off fluctuations; mean age 61.5 years (58-65); mean disease duration 13.5 years (5-18)	daily off periods, UPDRS motor scores	Mean reduction in daily off periods 94.5%; speed, quality, duration comparable to subcutaneous; no loss of effect or increased dyskinesia Mean required dose 5.75 mg	Slight vestibulitis in one patient
^24^	Intranasal transplantation of ANGE-S003 human neural stem cells (1.5 million, 5 million, or 15 million cells, four times)	12-month, single-center, open-label, dose-escalation phase 1 study	Eighteen patients with advanced PD	Safety and tolerability; efficacy via MDS-UPDRS total score, onset/duration of action	14 AEs in 7 patients, no serious AEs related to ANGE-S003; significant MDS-UPDRS improvement (mean reduction 19.9 points at month 6, *P* < 0.001) sustained to month 12; no dose-response relationship	Seven patients experienced 14 AEs. no safety concerns, no mass formation on brain MRI
^28^	rhEPO SC 40,000 IU weekly (5 weeks); neuroEPO intranasal 1 mg weekly (5 weeks) after 6 month follow up	Two clinical trials (open label)	Study 1: 10 PD patients; Study 2: 26 PD patient aged 60-66 years; Hoehn and Yahr stages 1-2	Cognitive function (global cognitive functioning, executive function, memory, DRS)	Positive response in cognitive functions in both studies (*P* < 0.05) compared to baseline	No serious adverse effect mentioned
^27^	Intranasal NeuroEPO (1 mL, 1 mg/mL) once weekly for 5 weeks	Monocentric, randomized, placebo-controlled, double-blind trial	26 PD patients; stages 1-2 on Hoehn and Yahr Scale; NeuroEPO (n = 15), placebo (n = 11)	Short-term tolerance (adverse events, blood pressure, hematological variables)	No significant difference in adverse events (20.0% NeuroEPO vs. 9.1% placebo, *P* = 0.22); NeuroEPO: 3 nauseas, 1 vomiting; placebo: 1 polyuria, 1 nasal irritation; all mild, brief, no treatment required	Three Nausea and one vomiting possibly due to patients positioning
^29^	Intranasal NeuroEPO	Double-blind safety trial	26 PD patients; NeuroEPO (n = 15), placebo (n = 11)	Cognitive scores (Mini Mental State Examination (MMSE) and DRS); quantitative electroencephalogram (qEEG) changes	Cognitive improvement correlated with qEEG (r = 0.97); cognition positively dependent on dose (*P* = 0.006) and qEEG (*P* < 0.0001); 66% of cognitive effect mediated by qEEG (*P* = 0.0001)	Mild and likely related to patient positioning while administration in three patients
^10^	40 IU Intranasal insulin (INI) once daily for 4 weeks	Randomized, double-blinded, placebo-controlled pilot study	15 PD patients (14 completed) 8 receiving INI six placebo and 1 MSA patient	Phonological and Semantic Verbal Test (FAS) score, motor performance (UPDRS, Hoehn and Yahr), Beck Depression Inventory (BDI) and gait test	INI group showed improvement in FAS score (41 ± 8.2 vs. 30.8 ± 7.1, *P* = 0.02), improved UPDRS-Motor (*P* = 0.02), improved Hoehn and Yahr (*P* = 0.04) and MSA patient was stable; placebo group unchanged	Well tolerated; no hypoglycemic episodes or serious adverse events
^31^	Intranasal rivastigmine 3.126 mg nasal vs. 1 mg intra venous	Phase 1 study	8 Healthy elderly individuals	Bioavailability (F, Cmax, Tmax, AUC) and safety	Nasal rivastigmine spray showed good safety and tolerability. 62% bioavailability. rapid absorption (Tmax ~1.1 h). exposure was comparable to 6–9.7 mg/day oral dosing or a 10 cm^2^ patch.	Two of the five adverse events reported were plausibly related to treatment (one mild nasal congestion and one mild, red, itchy stomach rash; both recovered within 12 hours). remaining three adverse events were mild cough, drowsiness and rash.
^33^	25 µg single dose Intranasal cholecystokinin-8	Randomized, placebo-controlled, crossover study	13 PD patients and thirteen healthy controls	Auditory evoked potentials (AEPs) (N2/P3 latencies/amplitudes), motor performance and UPDRS-III	PD patients showed delayed N2 and P3 AEP components after CCK-8 (*P* < 0.05), controls showed enhanced P3, shortened N2/P3 latencies (*P* < 0.05); no motor performance change in either group	No serious adverse effect reported
^35^	300 mg per day or 600 mg per day Intranasal glutathione (in)GSH in three divided doses for 3 months	Randomized, double-blind, placebo-controlled Phase I/IIa study	30 (28 completed) PD patients. modified Hoehn & Yahr stage ≤ 3; age ≥ 21; diagnosed within 10 years	Safety, tolerability (adverse events, compliance, withdrawals) and UPDRS trends	No substantial differences in adverse events between groups. all groups met tolerability criteria	Safe and well tolerated. no significant adverse events reported
^36^	Intranasal glutathione (in)GSH	Survey-based study	70 (of which 66) used respondents from 300 surveyed (response rate was 23.3%). PD (n = 7), multiple chemical sensitivity, allergies/sinusitis, Lyme disease, fatigue, others	Patients reported tolerability, adverse events, and health benefits	78.8% reported positive experience; 62.1% reported health benefits; 12.1% reported adverse effects	86% found spray comfortable and easy to administer; 12.1% experienced adverse effects
^37^	200 mg single dose Intranasal glutathione (in)GSH administration	Open-label study	Fifteen participants with mid-stage PD	GSH Concentration in CNS with proton magnetic resonance spectroscopy(H-MRS)	Significant increase in brain GSH relative to baseline (*P* < 0.001); all time points elevated after 8 min (*P* < 0.01)	Single paresthesia resolved within 1 h.
^38^	100 mg or 200 mg Intranasal glutathione (in)GSH thrice a day for 3 months	Double-blind, placebo-controlled Phase IIb study	45(40 completed) PD patients; Hoehn & Yahr stage 1–3	UPDRS total and motor sub score changes, Non-Motor Symptom Score (NMSS), magnetic resonance spectroscopy (MRS)	High-dose group: UPDRS total improved by 4.6 points (*P* = 0.0025), motor sub score by 2.2 points (*P* = 0.0485); no superiority over placebo	One cardiomyopathy case in high-dose group
^41^	Sublingual ipratropium bromide spray (1–2 sprays, max four times per day) for 2 weeks	Randomized, double-blind, placebo-controlled crossover study	17 PD patients with sialorrhea (15 completed)	Saliva weight (dental rolls), UPDRS salivation, diaries	No significant effect on saliva weight; mild effect on subjective sialorrhea measures	Well tolerated with no significant adverse events
^43^	Intranasal desmopressin (DDAVP), 20 μg bedtime for 1 week each post-baseline	Open-label study	8 (5 completed) PD patients with nocturnal polyuria; five completed trials	Nocturnal voids; safety (electrolytes, blood pressure)	Significant reduction in nocturnal voids in all completers	One patient developed hyponatremia and confusion, resolved post-discontinuation; two dropped out due to compliance issues

 Limitations include small sample sizes (e.g., n = 4–45) and open-label or pilot designs in many studies, particularly for insulin, glutathione, and neural stem cells, which restricted statistical power.^10,23,24,44^ Variability in apomorphine dosage (0.2–5.75 mg) and delivery devices across studies affect reproducibility.^9,19,20^ The lack of long-term data and standardized protocols limit clinical applicability. Cholecystokinin’s negative cognitive effects highlight the need for cautious evaluation of non-dopaminergic agents.^33^ While adverse events were generally mild (e.g., nasal irritation), rare serious events, such as cardiomyopathy with glutathione, necessitate monitoring.^44^

 Theoretically, these findings confirm intranasal delivery’s potential to enhance CNS targeting and reduced systemic exposure possibly by nose to brain pathways. Practically, nasal apomorphine and neural stem cells could improve motor fluctuation management, while insulin, glutathione and NeuroEPO target non-motor symptoms. Future research should focus on larger, multicenter, randomized controlled trials to validate efficacy. There are two clinical trials for insulin (NCT04687878, NCT04251585) helping to find out its clinical efficacy. Standardizing dosing and delivery devices, using devices with new technologies like ultrasound^45^, Electric-Guided Delivery of Charged Particles^46^ and conjugation with specific ligands^47^, using other dosage forms except solutions like gels^13,48^, mucoadhesive agents^49^ and powders^50^, using new formulations such as nanoparticles^51,52^ and liposomes^53^, alongside long-term safety and efficacy studies, is essential for clinical translation. This review underscores intranasal administration potential to transform PD’s traditional treatment. Evidence suggests that intranasal interventions can overcome pharmacokinetic challenges in PD treatment, warranting further investigation to improve therapeutic outcomes and patient quality of life.

## Conclusion

 This review evaluated the clinical efficacy, safety, and tolerability of intranasal interventions for Parkinson’s disease treatment, focusing apomorphine, insulin, glutathione, erythropoietin, human neural stem cells, ipratropium bromide, cholecystokinin, rivastigmine, and desmopressin. Intranasal apomorphine showed rapid onset of action and significant motor improvements, with UPDRS motor score reduction and reduction of daily ‘off’ periods, offering a noninvasive alternative for subcutaneous delivery to manage ‘off’ episodes. Intranasal insulin improved verbal fluency and UPDRS motor scores. Intranasal administration of glutathione increased CNS levels with UPDRS improvement. NeuroEPO enhanced cognitive outcomes. human neural stem cells reduced MDS-UPDRS scores. Desmopressin significantly reduced nocturnal voids. ipratropium bromide and cholecystokinin showed limited or no significant motor benefits, even cholecystokinin delayed cognitive processing. Adverse events were primarily mild and transient such as nasal or throat irritation across studies, supporting its good tolerability. These findings indicate that intranasal delivery can overcome limitations of conventional PD treatments, such as delayed onset, variable absorption and reducing systemic effects. Further research is needed to confirm efficacy, optimization and evaluation of new formulations.

## Competing Interests

 The author declares no conflict of interest

## Ethical Approval

 This approval was not required for this study, as it is review article based on previously published clinical studies.
